# The Degree of Programmed Death-Ligand 1 (PD-L1) Positivity as a Determinant of Outcomes in Metastatic Triple-Negative Breast Cancer Treated With First-Line Immune Checkpoint Inhibitors

**DOI:** 10.7759/cureus.21065

**Published:** 2022-01-09

**Authors:** Lorenzo Di Spazio, Melania Rivano, Luca Cancanelli, Marco Chiumente, Daniele Mengato, Andrea Messori

**Affiliations:** 1 Hospital Pharmacy, S.Chiara Hospital, Trento, ITA; 2 Clinical Oncology Pharmacy, A. Businco Hospital, Cagliari, ITA; 3 Hospital Pharmacy, Azienda Ulss 2 Marca Trevigiana, Treviso, ITA; 4 Scientific Direction, Italian Society for Clinical Pharmacy and Therapeutics, Milano, ITA; 5 Hospital Pharmacy, Azienda Ospedale Università di Padova, Padova, ITA; 6 Health Technology Assessment (HTA) Unit, Regione Toscana, Firenze, ITA

**Keywords:** restricted mean survival time, overall survival, pd-1 inhibitors, pd-l1 inhibitors, metastatic triple-negative breast cancer

## Abstract

In metastatic triple-negative breast cancer (TNBC), the efficacy of immune checkpoint inhibitors (ICIs) in combination with chemotherapy has been demonstrated in randomized clinical trials (RCTs). Despite this, an indirect comparison is not yet available. Reconstruction of individual patient data from Kaplan-Meier curves allows the indirect comparison of different treatments. We analyzed six overall survival (OS) curves from three RCTs. In patients with ≥1% positivity, atezolizumab was found to determine a significantly better OS than pembrolizumab. As regards pembrolizumab, adopting a threshold of PD-L1 positivity ≥10% (as opposed to ≥1%) improved median survival to a remarkable extent (23.0 vs 15.5 months).

## Introduction and background

Triple-negative breast cancer (TNBC) accounts for approximately 15% of all breast cancer and is characterized by the absence of expression of both estrogen (ER) and progesterone (PgR) receptors as well as of human epidermal growth factor receptor 2 (HER2). This disease condition is associated with a poor prognosis [1].

The options for systemic treatment of TNBC are limited. The disease is not sensitive to endocrine therapy and molecular-targeted therapy [2], and so surgery and systemic chemotherapy are the main therapeutic approaches. Adjuvant treatment always includes anthracycline and/or paclitaxel in early TNBC, but this treatment is generally ineffective after recurrence or metastasis.

Programmed death ligand 1 (PD-L1) is highly expressed in some breast cancer subtypes, especially in TNBC, where PD-L1 expression has been reported to be approximately 20% [3]. Moreover, PD-L1 expression can be upregulated by conventional chemo- and radiotherapies [4].

The most recent studies and clinical trials in this area have focused on the possible role of chemotherapy combined with programmed cell death protein 1 (PD-1) or PD-L1 inhibitors [5-7]. Pembrolizumab and nivolumab are the most common monoclonal antibodies targeting PD-1, while atezolizumab, durvalumab, and avelumab are targeting PD-L1 [8].

In the first-line treatment of metastatic TNBC, the final results of KEYNOTE-355 [5] have shown that pembrolizumab in association with paclitaxel improves overall survival (OS) as compared with paclitaxel alone (median, 23.0 vs 16.1 months; hazard ratio [HR], 0.73 with 95% confidence interval [CI] of 0.55 to 0.95). These results refer to the subpopulation with positivity ≥10% (220 vs 103 patients). The final results of KEYNOTE-355 [5] include also the survival data for the subpopulation with positivity ≥1%. After the release of the final findings from the KEYNOTE-355, the European Community (EC), through the European Medicines Agency (EMA), has approved pembrolizumab for this clinical indication in patients with positivity ≥10% [9].

Atezolizumab has also been tested for the same clinical indication: two trials have been conducted (IM-PASSION-130 [6] and IM-PASSION-131 [7]) that enrolled patients who met a threshold of PD-L1-positivity of ≥1%.

All in all, according to the criterion of positivity ≥1%, three trials on immune checkpoint inhibitors (KEYNOTE-355 [5], IM-PASSION-130 [6], and IM-PASSION-131 [7]) are currently available and can therefore be compared to assess the relative effectiveness of these treatments in this subset of patients.

## Review

Literature search and selection of pertinent clinical trials

Our review retrospectively examined the survival findings reported in the three abovementioned trials (i.e. KEYNOTE-355 [5], IM-PASSION-130 [6], and IM-PASSION-131 [7]). In more detail, we analyzed six cohorts with positivity ≥1% (namely, two cohorts treated with atezolizumab plus paclitaxel [6,7], one with pembrolizumab plus paclitaxel [5], and three cohorts treated with paclitaxel alone [5-7]); paclitaxel alone represented the treatment given to the control groups of the three trials. Table 1 summarizes the characteristics of these six cohorts. In our analysis, we indirectly compared these cohorts with one another according to the end-point of OS.

**Table 1:** Treatments given as first-line in three randomized trials. *This should be assumed to be positivity of ≥1%. **Reference [5] also reports the overall survival determined in the population with CPS≥10% that included 220 patients given pembrolizumab and 103 controls; events were 155 and 84, respectively. Abbreviations: n, number of events; N, the total number of patients; RMST, restricted mean survival time; CPS, Combined Positive Score.

Trial	First author, year of publication	PD(L)1 positivity	Treatment group (n/N)	Control group (n/N)	Survival curve reported in the original publication
IM-PASSION-130	Emens, 2021 [6]	PD-L1 positive population*	Atezolizumab plus nab-paclitaxel (120/185)	Placebo plus nab-paclitaxel (139/184)	Figure 1B
IM-PASSION-131	Miles, 2021 [7]	PD-L1 positive population*	Atezolizumab plus paclitaxel (72/191)	Paclitaxel (51/101)	Figure 3A
KEYNOTE-355	Rugo, 2021** [5]	CPS≥1%	Pembrolizumab plus chemotherapy (336/425)	Chemotherapy (177/211)	URL on the Internet (see [5])

Statistical analysis

In analyzing each of these cohorts, we applied a validated technique of individual patient data reconstruction, the Shiny method [10]. According to the Shiny method, the graph of each of the six Kaplan-Meier OS curves was digitalized and converted into x-y data pairs using Webplotdigitizer [11]. Then, the Shiny package (Version: 1.2.2.0; subprogram “Reconstruct Individual Patient Data”; URL https://www.trialdesign.org/one-page-shell.html#IPDfromKM [10]) was used to reconstruct patient-level data on the basis of x-y data pairs of the curve, total number of enrolled patients, and total number of events. Finally, the pooled survival curves were generated from the reconstructed patient-level data and evaluated through Cox statistics; for this purpose, we used three packages (“coxph,” “survfit,” and “ggsurvplot”) under the R-platform [12]. The values of restricted mean survival time (RMST) were also estimated from these six curves using the “survRM2” package of the R-platform. The generation of pooled survival curves from reconstructed individual patient data has recently been shown to be a simple but efficient alternative to survival meta-analysis [13-15].

Pooled survival information determined from reconstructed individual patient data

**Figure 1:**
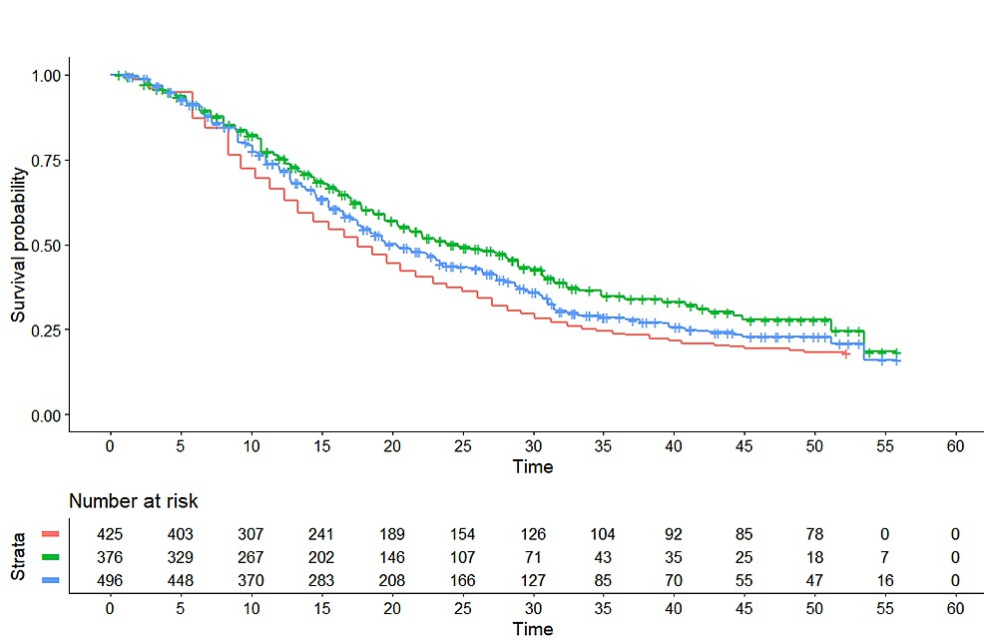
Kaplan-Meier curves from reconstructed patient-level data. These three survival curves refer to PD-L1 positive patients and were obtained by reconstruction of individual patient data from two trials for atezolizumab (IM-PASSION-130 and IM-PASSION-131) and one for pembrolizumab (KEYNOTE-355). The cohorts given atezolizumab consisted of 376 patients (IM-PASSION-130, N=185; IM-PASSION-131, N=191). Pembrolizumab included 425 patients. The control groups of the three trials, pooled together, included 496 patients. End-point of the curves: overall survival. Symbols: atezolizumab in green, pembrolizumab in red, controls in blue; time expressed in months.

The pooled Kaplan-Meier curves generated by our analysis are shown in Figure 1.

Atezolizumab showed a significantly better OS compared with pembrolizumab (HR, 0.73, 95%CI of 0.61 to 0.87, p<0.001; median, 20.4 vs 15.5 months). Surprisingly enough, pembrolizumab fared worse than the controls, this difference being at limits of statistical significance (HR, 1.16, 95%CI of 1.00 to 1.35, p=0.05; median, 15.5 vs 18.4 months). Atezolizumab showed better survival than controls, though at limits of statistical significance (HR, 0.85; 95%CI, 0.67 to 1.07; median, 20.4 vs 18.4 months; p=0.06). In a separate analysis (data not shown), the survival pattern of control groups was found to be virtually identical across the three trials.

The values of RMST (along with pairwise differences) were estimated to be the following: 

- atezolizumab (two cohorts) vs pembrolizumab (one cohort): 27.6 months (95%CI, 25.6 to 29.6) vs 22.9 (95%CI, 21.3 to 24.4); difference, 4.7 months (95%CI, 2.2 to 7.3), p<0.001;

- atezolizumab (two cohorts) vs controls (three cohorts), 27.6 months (95%CI, 25.6 to 29.7) vs 25.2 (95%CI, 23.6 to 26.7), difference, 2.4 months (95%CI, -0.14 to 5.0), p=0.063;

- pembrolizumab (one cohort) vs controls (three cohorts): 22.9 months (95%CI, 21.3 to 24.4) vs 25.2 (95%CI, 23.6 to 26.7); difference, -2.3 months (95%CI, -4.5 to 0.14), p=0.037.

The above p-values are those estimated by the “survRM2” package under the R-platform; the truncation time of each analysis was set, in accordance with the Kaplan-Meier curves of the various cohorts, at 50 months.

Interpretation of survival findings

The main finding arising from our analysis is the longer OS found for atezolizumab compared with pembrolizumab. Although this difference showed a clear statistical significance, caution is needed in its interpretation owing to the indirect nature of our comparisons. As a result, the superiority of atezolizumab over pembrolizumab should be viewed as one hypothesis requiring a more robust confirmation based on further data. This hypothesis, however, deserves to be reported to the scientific community as a useful piece of information in the debate on the effectiveness of immune checkpoint inhibitors in metastatic TNBC.

Another point emerging from our analysis is that the degree of PD-L1 positivity seems to be a strong determinant of survival. In the case of pembrolizumab, adopting a threshold of PD-L1 positivity ≥10% (as opposed to ≥1%) improved median survival from 15.5 to 23.0 months; in the case of atezolizumab, a similar effect is likely to occur, even though specific data based on the ≥10% threshold have not been published. Therefore, as regards atezolizumab, one further analysis for the IM-passion-131 trial [16,17] would be needed in which OS is selectively determined in the subpopulation with a combined positive score (CPS) of PD-L1-positivity ≥10%. Atezolizumab in fact might maximize its benefit in this specific patient subpopulation, but the analysis of this patient subgroup has yet not been reported in the published literature.

The present analysis has strengths and weaknesses. One strength is the excellent performance of the Shiny method [10,13-15] in reconstructing individual patient data from published Kaplan-Meier survival curves. The quality of our reconstruction of patient-level data was also confirmed by the concordance of results based on original values of HR with those based on reconstructed values of HRs and RMST. Another strength is that, in general, this strategy of evidence analysis facilitates the task of carrying out indirect comparisons on therapeutic issues suggested by the recent literature. This holds true particularly when direct comparisons based on a “real” trial are not available and are also unlikely to become available. As already pointed out, data obtained in this way may contribute to the clinical debate on the comparative effectiveness of different and competitor drugs.

As regards weaknesses of our analysis, the most obvious one is the indirect nature of our comparisons between pembrolizumab+paclitaxel *vs.* atezolizumab+paclitaxel *vs.* paclitaxel alone. This limitation is intrinsic to the procedure of reconstructing patient-level data from Kaplan-Meier graphs in a context where “real” individual patient data are not available and the only covariate for statistical analysis is the treatment received by the patient. In summary, one should keep in mind that this type of comparison, which has by definition a retrospective design, does not take into account the contribution of unavoidable intrinsic differences in the patients' cohorts treated in different studies.

Another source of uncertainty in our analysis lies in the different methods of PD-L1 assay employed in atezolizumab vs pembrolizumab trials; however, despite some objective differences in these assays, they likely had a marginal effect on the results of the three trials [18].

A final note is needed about sacituzumab govitecan. This agent is a new antibody-drug conjugate that has proved to be effective in patients with metastatic TNBC [19]. However, while our analysis was focused selectively on first-line treatments, sacituzumab has thus far been tested only after two or more previous chemotherapy regimens. As a result, this new drug still needs to be evaluated in earlier lines, and especially as first-line therapy.

## Conclusions

Our analysis suggests a difference in effectiveness between atezolizumab and pembrolizumab. This difference has a remarkable clinical relevance (gain: 4.9 months according to medians and 4.7 months according to RMST) and is supported by sound statistical significance (p<0.001). Furthermore, the two trials evaluating atezolizumab (IM-PASSION-130 and IM-PASSION-131) showed no meaningful differences in inclusion criteria compared pembrolizumab (KEYNOTE-355), and so the difference in enrolled patient populations is unlikely to explain the different survival outcomes. More importantly, the control groups of the two trials of atezolizumab showed a very similar survival pattern compared with the controls of pembrolizumab.

Despite this, owing to the indirect nature of our comparison, we conclude that our result should be viewed as preliminary and therefore will need confirmation based on a direct comparison.

## References

[1] Pareja F, Reis-Filho JS (2018). Triple-negative breast cancers - a panoply of cancer types. Nat Rev Clin Oncol.

[2] Foulkes WD, Smith IE, Reis-Filho JS (2010). Triple-negative breast cancer. N Engl J Med.

[3] Mittendorf EA, Philips AV, Meric-Bernstam F (2014). PD-L1 expression in triple-negative breast cancer. Cancer Immunol Res.

[4] Peng J, Hamanishi J, Matsumura N (2015). Chemotherapy induces programmed cell death-ligand 1 overexpression via the nuclear factor-κB to foster an immunosuppressive tumor microenvironment in ovarian cancer. Cancer Res.

[5] Rugo H (2021). Final results of KEYNOTE-355 (LBA16): a randomized, double-blind, phase-3 study of pembrolizumab + chemotherapy vs placebo + chemotherapy for previously untreated locally recurrent inoperable or metastatic triple negative breast cancer. Oncology PRO - ESMO.

[6] Emens LA, Adams S, Barrios CH (2021). First-line atezolizumab plus nab-paclitaxel for unresectable, locally advanced, or metastatic triple-negative breast cancer: IMpassion130 final overall survival analysis. Ann Oncol.

[7] Miles D, Gligorov J, André F (2021). Primary results from IMpassion131, a double-blind, placebo-controlled, randomised phase III trial of first-line paclitaxel with or without atezolizumab for unresectable locally advanced/metastatic triple-negative breast cancer. Ann Oncol.

[8] Li Y, Miao W, He D, Wang S, Lou J, Jiang Y, Wang S (2021). Recent progress on immunotherapy for breast cancer: tumor microenvironment, nanotechnology and more. Front Bioeng Biotechnol.

[9] (2021). Merck Press release. KEYTRUDA Is Now Approved in Combination With Chemotherapy as First-Line Treatment for Patients With Locally Recurrent Unresectable or Metastatic TNBC Whose Tumors Express PD-L1 (CPS ≥10) and Who Have Not Received Prior Chemotherapy for Metastatic Disease. https://www.merck.com/news/european-commission-approves-mercks-keytruda-pembrolizumab-plus-chemotherapy-as-treatment-for-certain-patients-with-locally-recurrent-unresectable-or-metastatic-triple-negative-breast/.

[10] Liu N, Zhou Y, Lee JJ (2021). IPDfromKM: reconstruct individual patient data from published Kaplan-Meier survival curves. BMC Med Res Methodol.

[11] Rohatgi A (2021). Webplotdigitizer. Version 4.5. https://automeris.io/WebPlotDigitizerc.2021.05.801.

[12] (2021). R: a language and environment for statistical computing. http://www.r-project.org.

[13] Messori A (2021). Synthetizing published evidence on survival by reconstruction of patient-level data and generation of a multi-trial Kaplan-Meier curve. Cureus.

[14] Cancanelli L, Rivano M, Di Spazio L (2021). Efficacy of immune checkpoint inhibitors in patients with mismatch repair-deficient or microsatellite instability-high metastatic colorectal cancer: analysis of three phase-II trials. Cureus.

[15] Messori A, Rivano M, Mengato D, Cancanelli L, Di Spazio L, Chiumente M (2022). A preliminary estimate of survival gain and cost-effectiveness of CAR-T in adult patients with acute lymphoblastic leukaemia [PREPRINT]. Leukemia & Lymphoma.

[16] (2021). A study of atezolizumab and paclitaxel versus placebo and paclitaxel in participants with previously untreated locally advanced or metastatic triple negative breast cancer (TNBC). https://clinicaltrials.gov/ct2/show/study/NCT03125902.

[17] Cortes J, Cescon DW, Rugo HS (2020). Pembrolizumab plus chemotherapy versus placebo plus chemotherapy for previously untreated locally recurrent inoperable or metastatic triple-negative breast cancer (KEYNOTE- 355): a randomised, placebo-controlled, double-blind, phase 3 clinical trial. Lancet.

[18] Huang X, Ding Q, Guo H (2021). Comparison of three FDA-approved diagnostic immunohistochemistry assays of PD-L1 in triple-negative breast carcinoma. Hum Pathol.

[19] Bardia A, Hurvitz SA, Tolaney SM (2021). Sacituzumab govitecan in metastatic triple-negative breast cancer. N Engl J Med.

